# Emergence and Pathogenicity of a Novel Goose Adenovirus Type 4 Strain JA2485: Insights for Poultry Disease Control

**DOI:** 10.3390/v17121560

**Published:** 2025-11-28

**Authors:** Bingjie Li, Jingjing Chang, Xiaoyang Cao, Wenwen Zhou, Lin Liu, Wenming Gao, Zongmei Huang, Jingrui Liu, Xiaojie Zhou, Yuman Liu, Yapeng Song, Xinsheng Li

**Affiliations:** College of Veterinary Medicine, Henan Agricultural University, Zhengzhou 450002, China

**Keywords:** Goose Adenovirus Type 4, genetic evolution, pathogenicity

## Abstract

In recent years, the prevalence and variation of goose adenoviruses, especially Goose Adenovirus Type 4 (GoAdV-4), have threatened waterfowl farming, while their genetic evolution and pathogenic mechanisms remain unelucidated. In May 2024, a novel GoAdV-4 strain (JA2485) was isolated from diseased Sanhua geese in Jian City, Jiangxi Province, China. It propagated in 11–13-day-old goose embryos, with a 50% embryo infectious dose (EID_50_) of 10^2.2^/0.1 mL. Whole-genome sequencing (GenBank Accession No. PQ152938) showed its genome is 43,030 base pairs long, containing 33 protein-coding regions and 2 fiber genes. Phylogenetic analysis revealed JA2485 is closely related to the Hungarian P29 strain and Chinese CH-FJZZ-202201 strain (nucleotide similarity: 94–96.8%), but clusters in a different branch from other avian adenoviruses. In pathogenicity tests on 1-day-old Sanhua geese, both subcutaneous injection and oral inoculation groups had 50% mortality. Infected geese showed weight loss, depression, reduced appetite, increased recumbency, and even paralysis in severe cases. Post mortem examination revealed hepatic rounded margins, yellowing, focal hemorrhages, and renal hemorrhagic lesions. Notably, viral loads were highest in the liver, duodenum, and cloacal swabs, suggesting fecal transmission. This study provides a key basis for clarifying GoAdV-4’s evolutionary characteristics and pathogenic mechanisms, and formulating targeted prevention strategies.

## 1. Introduction

Adenoviruses are double-stranded DNA viruses that are non-enveloped and exhibit an icosahedral morphology, with a diameter ranging from 70 to 100 nanometers [1]. According to the International Committee on Taxonomy of Viruses (ICTV), the family adenoviridae is classified into six genera, aviadenovirus, mastadenovirus, siadenovirus, atadenovirus and ichtadenovirus [2]. Fowl adenoviruses are further categorized into 15 species based on their host specificity, including Fowl avianadenoviruses (FAdV) A−E, Duck avianadenoviruses (DAdV) A and B, Goose avianadenoviruses (GoAdV) A, Pigeon avianadenoviruses (PiAdV) A and B, Parrot avianadenoviruses (PsAdV) B, Turkey Adenovirus (TAdV) A-C, Falcon avianadenoviruses A [3]. Among these, FAdV, DAdV and GoAdV are capable of infecting domestic waterfowl. The primary clinical manifestations include reduced appetite, lethargy and decreased egg production, with severe cases potentially leading to mortality. Currently, numerous FAdV strains originating from duck and goose have been isolated from domestic waterfowl, whereas isolations of various subtypes of DAdV and GoAdV remain relatively limited.

GoAdV was initially identified in Hungarian goose flocks in 1967 [4]. In 1984, seven strains of goose adenovirus were isolated from the livers and intestines of diseased geese in Hungary. In the neutralization test, these adenoviruses did not exhibit cross-reactivity with 11 poultry adenoviruses serotypes, or 2 TAdV serotypes and 2 DAdV serotypes. Subsequently, based on endonucleases DNA cleavage patterns, these 7 isolates were classified into 3 distinct serotypes: GoAdV-1, GoAdV-2 and GoAdV-3 [5]. In 2012, a comprehensive phylogenetic analysis of the GoAdV strain P29, isolated from deceased geese in Hungary, revealed that this strain formed a monophyletic lineage within the genus Avianadenovirus. Consequently, it was designated as GoAdV-4 [6]. In 2022, comparative genomic analyses across avian adenovirus demonstrated that the G + C content of P29 strain was significantly different from that of FAdV, suggesting substantial genetic divergence between GoAdV and FAdV [7]. In 2023, the GoAdV-4 CH-FJZZ-202201 strain was first isolated from sick and dead geese in Fujian Province, China, and successfully propagaed in goose embryo fibroblasts (GEF) [8]. Since its initial discovery, research on the serological and genetic characteristics of goose adenovirus has progressed steadily; however, its pathogenic characteristics and mechanism remain poorly understood.

In May 2024, a virus suspected to be Goose adenovirus serotype 4 (GoAdV-4) was isolated from Sanhua geese at a commercial goose farm located in Jian City, Jiangxi Province. Full-genome sequencing and phylogenetic analysis were Subsequently conducted on the isolate, which was designated as strain JA2485. The virus was propagated in goose embryos for isolation and culture, and its 50% embryo infectious dose (EID_50_) was determined. Following this, the viral strain was then used to experimentally infect 1-day-old Sanhua geese via both subcutaneous and oral inoculation routes. Mortality and morbidity of the infected goslings were systematically recorded. Histopathological changes in tissue samples were examined, and viral loads in pharyngeal and cloacal swabs as well as various organs were quantified. These findings contribute foundational insights into the ongoing evolutionary dynamics and pathogenic properties of goose adenovirus.

## 2. Materials and Methods

### 2.1. Sample Collection

Heart, liver and kidney tissues were collected from 4-week-old diseased Sanhua geese in poultry flocks located in Jiangxi Province, China, in May 2024. The estimated prevalence of the disease was 5%, with a daily mortality rate at the peak ranging from 6% to 8%.

### 2.2. Virus Isolation

Heart and liver tissues from diseased chickens were subjected to three freeze–thaw cycles. The resulting homogenate was centrifuged at 12,000× *g* at 4 °C for 10 min and then filtered through a 0.22 µm membrane filter (Millipore, Billerica, MA, USA). Total DNA and RNA were extracted separately from the homogenized tissues using a viral DNA/RNA kit (Vazyme, Nanjing, China). Potential avian pathogens were screened using polymerase chain reaction (PCR) (BIOER, Hangzhou, China). The detection panel included avian adenovirus (FAdV), goose astrovirus (GAst), goose parvovirus (GPV), goose reovirus (GRV), duck parvovirus (MDPV), avian influenza viruses H5, H7, and H9 (AIV), Newcastle disease virus (NDV), and infectious bursal Fabricius virus (IBDV). Specific primer sequences used in the PCR assays are listed in Table 1. The primers were synthesized by Shanghai Sangon Biotechnology Co., Shanghai, China. To isolate and purify the virus, 0.2 mL of the tissue supernatant was inoculated into the chorioallantoic membranes of 11~13-day-old goose embryos. Following at 37 °C for 120 h, the chorioallantoic membranes were harvested. The presence of the virus in the collected chorioallantoic fluid was confirmed by PCR, followed by passage through three consecutive generations in goose embryos. Subsequently, the EID_50_ of GoAdV infection was calculated using the Reed-Muench method [9]. The supernatant from the villous allantoic membrane of the third passage was serially diluted from 10^−1^ to 10^−5^ and inoculated into the goose embryos following the same procedure, with 5 goose eggs allocated for each dilution. In this study, the final EID_50_ value was determined as the average of three independent replicate experiments.

### 2.3. Genome Sequencing

To obtain the complete genome of the virus, 25 pairs of primers (Table 2) were designed to amplify overlapping genomic fragments, which together cover the entire viral genome. The virus strain isolated in this study was designated as JA2485. First, the targeted overlapping genomic fragments were subjected to PCR amplification to generate sufficient DNA templates. Subsequent sequencing of the amplified fragments was performed by Shanghai Sangon Biotechnology Co. Sequencing was conducted by the company using the Illumina HiSeq platform, with a sequencing depth of ≥50× for each amplified fragment to ensure the accuracy of base calling. After sequencing, all obtained valid sequences were assembled and concatenated into a continuous full-length viral genome using DNAMAN V6 software. After assembly with DNAMAN V6 software, the overlapping regions of each amplified fragment (each ≥200 bp in length) were analyzed using SnapGene 6.2.2 software. The results revealed no base conflicts or sequence gaps, and the continuity of the assembled sequence reached 100%. Finally, a schematic diagram of the complete viral genome sequence was constructed using SnapGene 6.2.2 software.

### 2.4. Phylogenetic Analysis

For phylogeny analysis of strain JA2485, nucleotide sequences from 19 adenoviruses of diverse avian origins were retrieved from the GenBank database. Evolutionary relationships based on the complete genome and major structural proteins were inferred using the neighbor-joining method and the Kimura two-parameter method with bootstrap analysis (1000 replicates). Evolutionary distances were calculated using the pairwise distance approach under the maximum composite likelihood model. Phylogenetic trees were visualized using MEGA v7.0 software. Additionally, amino acid sequence identity was analyzed through MegAlign 17.3.1 software.

### 2.5. Pathogenicity Experiment

Forty-eight unvaccinated, seronegative against GoAdV-4, 1-day-old healthy Sanhua geese were randomly assigned to three groups: the subcutaneous injection group (*n* = 16), the oral infection group (*n* = 16), and the negative control group (*n =* 16). The goslings in the two experimental groups were infected with 0.4 mL of a 20% tissue supernatant suspension (viral copy number: 2.5 × 10^6^ copies/μL), whereas the control group received an equivalent volume of sterile phosphate-buffered saline (PBS). All goslings were housed in independent negative pressure isolators (Suzhou Purification Equipment Co., Ltd., Suzhou, China) and provided with ad libitum access to feed and water. Clinical symptoms and body weight changes were systematically monitored and recorded over a 14-day period.

### 2.6. Quantitative PCR (qPCR)

To establish a fluorescence quantitative PCR (qPCR) assay for GoAdV, specific forward (5′-ACCTCGCTATCCTGCTCAGA-3′) and reverse (5′-ACCGCAGGTAGGTGCTAAAC-3′) primers were designed based on the conserved 52K gene of adenoviruses. The primers were synthesized by Shanghai Sangon Biotechnology Co., Shanghai, China. The total reaction volume for the qPCR was 25 μL, consisting of 12.5 μL of SYBR Green PCR Master Mix (Takara Bio Inc., Kusatsu, Japan), 0.5 μL each of forward and reverse primers (10 μmol/L), 2 μL of cDNA template, and 9.5 μL of nuclease-free water (Ambion, Austin, TX, USA). The thermal cycling conditions were as follows: initial pre-denature at 95 °C for 30 s, followed by 40 cycles of denaturation at 95 °C for 5 s and annealing/extension at 60 °C for 30 s. Standard curves were constructed using 10^−3^~10^−8^ serial dilution of plasmid DNA, with the threshold cycle (Ct) value plotted against the logarithm of the viral copy number via linear regression analysis.

### 2.7. Viral Load

Oropharyngeal and anorectal swabs were collected from goslings in the two experimental groups at 1, 3, 5, 7, 9, 11 and 13 day-post-infection (dpi), respectively, and the viral load was quantified by qPCR. The swabs were immersed in 1.0 mL of PBS buffer and subjected to two freeze–thaw cycles. The resulting swab suspensions were stored at −80 °C prior to DNA extraction and subsequent analyses. Three geese from each group were euthanized at 4 and 14 dpi, respectively. Fresh tissues samples, including heart, liver, spleen, lung, kidney, thymus, bursa of Fabricius, small intestine, duodenum, muscle and glandular stomach, were collected and divided into two portions. One portion was used for qPCR to detect the viral load of each organ, and the other was utilized for histopathological sections preparation.

### 2.8. Histopathology

Tissues collected for histopathological examination were fixed in 4% formaldehyde solution (Sigma-Aldrich, St. Louis, MO, USA), embedded in paraffin (Leica Biosystems, Wetzlar, Germany), sectioned at a thickness of 4–6 μm, and stained with hematoxylin and eosin (H&E) (Abcam, Cambridge, UK) following standard histological protocols. All experimentally infected goslings were humanely euthanized 14 days post-infection (dpi), and histopathological alterations in the organs were examined under an optical microscope (Nikon, Tokyo, Japan). Histopathological lesions were scored using the following criteria [10]: 0 points (no lesion): intact organ structure, no inflammatory cell infiltration, and no parenchymal cell necrosis/degeneration; 1 point (mild): scattered infiltration of a small number of inflammatory cells, no parenchymal cell necrosis, and essentially normal organ structure; 2 points (moderate): focal aggregation of inflammatory cells, accompanied by a small amount of parenchymal cell necrosis/degeneration (e.g., focal hepatocyte necrosis, thickened alveolar septa, swollen renal tubular epithelium), with locally blurred structure; 3 points (severe): massive clustering of inflammatory cells, extensive parenchymal cell necrosis/degeneration (e.g., extensive hepatic necrosis, pulmonary vacuolation, renal tubular necrosis), with severely disorganized structure.

### 2.9. Statistical Analyses

First, the normality of the experimental data was verified using the Shapiro–Wilk test, and the homogeneity of variances was confirmed via the Levene test. The experimental data were analyzed and visualized using GraphPad Prism 8.0.2. All experimental results represent the mean values of three independent replicates. Statistical significance was evaluated using Student *t*-test, where * *p* < 0.05 indicated a significant difference, ** *p* < 0.01 indicated a highly significant difference, and *** *p* < 0.001 indicated an extremely significant difference.

## 3. Results

### 3.1. Isolation and Identification of JA2485 Strain

In May 2024, a viral strain was isolated from Sanhua geese at a commercial goose farm in Jiangxi Province, China. The infected geese initially exhibit reduced feed intake, followed by clinical signs of depression, which ultimately resulted in mortality. Gross pathological examination revealed hepatomegaly with rounded and blunt margins, along with inclusion body hepatitis and kidneys enlargement. Amplification using avian adenovirus specific primers yielded a positive PCR band of the expected size (507 bp). Furthermore, Blast analysis demonstrated that the amplified fragment exhibited a high sequence similarity of 99.53% to the GoAdV-4 strain CH-FJZZ-202201, suggesting that this isolate was most likely GoAdV-4. Subsequently, the virus strain was inoculated into goose embryos for isolation and propagation. After three consecutive passages in goose embryos, the PCR identification consistently confirmed the presence of the virus. Concurrently, the EID_50_ of the virus in goose embryos was determined to be 10^2.2^/0.1 mL.

### 3.2. Genome Analysis

**Figure 1:**
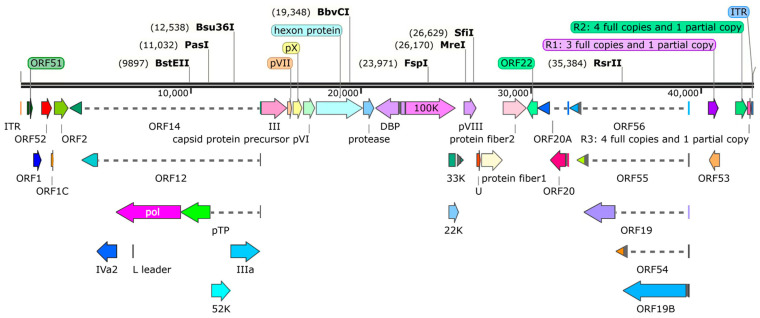
Genomic map of GoAdV-4 strain JA2485. Based on the whole-genome sequencing results of strain JA2485 (GenBank Accession No. PQ152938), SnapGene software was used to construct a schematic diagram of the linear double-stranded DNA genome of this strain. Open reading frames (ORFs), restriction endonuclease recognition sites, key genes and their encoded proteins, inverted terminal repeats (ITRs), copy numbers of R1–R3 repetitive regions were labeled, and a genome length scale was added. The dashed lines in the figure indicate the non-continuous coding regions of open reading frames (ORFs) or the intergenic spacer sequences between different genomic elements.

The complete genomic sequence of the isolate JA2485 has been deposited in GenBank under accession number PQ152938. According to the genetic map of JA2485, it conforms to the structural characteristics of linear double-stranded DNA (dsDNA) of adenovirus, without any signs of circularity or segmentation. The genome strain consists of 43,030 base pairs, with a G + C content of 44.4%. It encompasses 33 putative protein-coding regions, and there are palindrome repetitive sequences at both ends of the genome, among them the length of the inverted terminal repeat (ITR) sequence is 39 bp. This genome contains three core packaging proteins, namely pVI, pVII and pVIII. The genome encodes three major structural proteins characteristic of adenoviruses. Specifically, the hexon gene spans 2802 bp, and the penton base gene spans 1587 bp. Strain JA2485 contains two fiber genes: a shorter fiber 1 gene of 1281 bp and a longer fiber 2 gene of 1404 bp (Figure 1).

### 3.3. Phylogenetic Analysis

**Figure 2:**
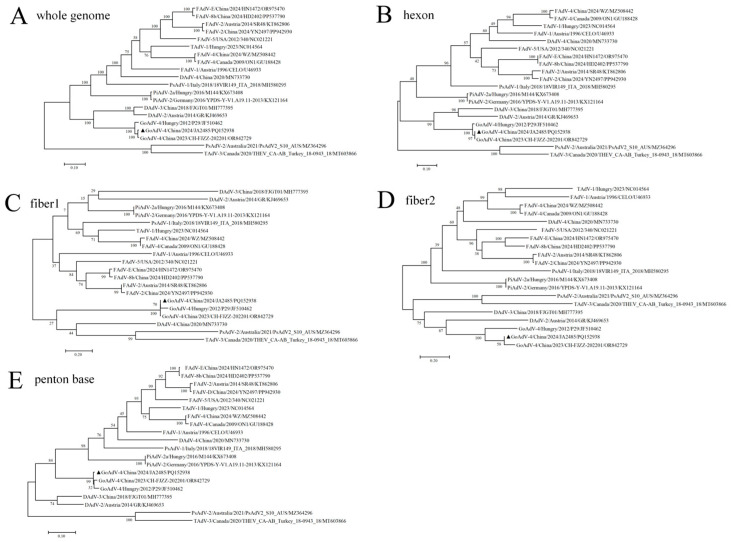
Phylogenetic analysis of GoAdV-4 strain JA2485. Nucleotide sequences of 19 different avian adenoviruses were retrieved from the GenBank database. Phylogenetic trees were constructed using the neighbor-joining method with the Kimura two-parameter model, based on the complete genome nucleotide sequence (**A**) and the amino acid sequences of the hexon (**B**), fiber1 (**C**), fiber2 (**D**), and penton base (**E**) proteins of strain JA2485. Strain JA2485 is marked with a black triangle in the figures. Bootstrap validation was performed with 1000 replicates, and the phylogenetic trees were visualized using MEGA v7.0 software.

Phylogenetic analysis based on the whole genome and other major structural proteins showed (Figure 2) that strain JA2485 formed distinct monophyletic clades with the strains GoAdV-4 P29 and CH-FJZZ-202201, with minimal genetic distances between them. This indicates a close evolutionary relationship and high genetic similarity among these three viral strains. The comparison of the entire genome sequences further revealed that the nucleotide similarity of the strain JA2485 to the Hungarian isolate P29 and the Chinese isolate CH-FJZZ-202201 was 94% and 96.8%, respectively. These findings support the classification of strain JA2485 as Goose adenovirus serotype 4 (GoAdV-4), species A. Further analysis of the phylogenetic trees of major structural proteins indicated that the three GoAdV strains exhibited very close genetic distances across all major structural proteins except fiber2. Sequence alignment revealed that the nucleotide similarities of the hexon gene between strain JA2485 and strains P29 and CH-FJZZ-202201 were 98.5% and 99.8%, respectively; those of the penton base gene were 96.7% and 97.7%, respectively; and those of the fiber1 gene were 95.9% and 99.5%, respectively. In contrast, the nucleotide similarities of the fiber2 gene were significantly lower, at 58% and 94.7%, respectively. These findings demonstrate that the fiber2 gene of JA2485 differs remarkably from that of P29, which may alter the function of the fiber2 protein and thereby result in differences in host cell receptor binding and infectivity between the two strains [11].

Except for the fiber 1 Phylogenetic analysis, strain JA2485 clustered closely with DAdV-2 strain GR and DAdV-3 strain 2018FJGT01 in the remaining phylogenetic trees. However, Phylogenetic analysis showed only 60.7% and 40.7% sequence identity with these two DAdV strains, respectively. This indicates a potential evolutionary link between the JA2485 strain and DAdV, although significant genetic divergence still exists. Strain JA2485 was phylogenetically distant from other reference strains derived from various avian species and isolates, forming a separate lineage. Sequence Phylogenetic analysis indicated identities of 46.7% with FAdV-A, 42.0% with FAdV-B, 41.3–42.3% with FAdV-C, 42.0–42.9% with FAdV-D, and 42.8–42.9% with FAdV-E. Additionaly, JA2485 exhibited 43.3–43.6% identity with PiAdV, 35.1–39.0% with PsAdV, and 35.8–40.0% with TAdV references strains. Collectively, these data indicate that strain JA2485 exhibits substantial genetic divergence and a distant evolutionary relationship compared to other known avian adenovirus types.

### 3.4. Pathogenicity

**Figure 3:**
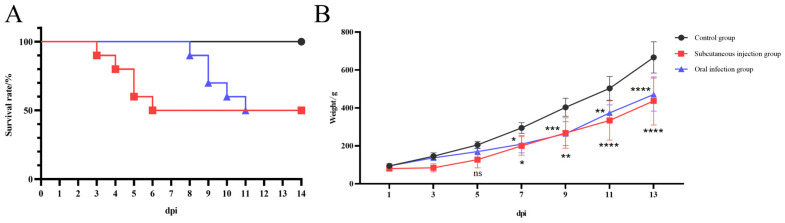
Health dynamics of 1-day-old Sanhua goslings infected with GoAdV-4 strain JA2485 (**A**,**B**). Forty-eight 1-day-old healthy Sanhua goslings, which were unvaccinated and seronegative for GoAdV-4, were randomly divided into three groups: subcutaneous injection group (*n* = 16), oral infection group (*n* = 16), and negative control group (*n* = 16, injected with sterile PBS). All goslings were housed in independent negative-pressure isolators. During the 14-day observation period, the survival status of goslings was recorded daily to generate the survival curve. The body weight of goslings was measured at 1, 3, 5, 7, 9, 11, and 13 days post-infection (dpi) to generate the body weight change curve. Data were presented as the mean ± standard deviation of three independent replicate experiments. * < 0.05, ** < 0.01, *** < 0.001, **** < 0.0001, indicating statistically significant differences between the groups.

Following subcutaneous injection and oral inoculation of the JA2485 strain in goslings, the mortality rate in both experimental groups reached 50% (Figure 3A). In the subcutaneous injection group, mortality primarily occurred between 3 and 6 dpi after infection, with one death on 3 dpi, one on 4 dpi, two on 5 dpi, and one on 6 dpi. The oral infection group exhibited a delayed onset of disease, with deaths occurring only between 8 and 11 dpi, one on 8 dpi, two on 9 dpi, one on 10 dpi, and one on 11 dpi. No mortality was observed in the control group, where goslings remained clinically healthy throughout the observation period.

Starting from 7 dpi, the average body weights of both the subcutaneous injection group and the oral infection group were significantly lower than that of the control group (Figure 3B). The most significant body weight loss in the two experimental groups was observed at 13 dpi. Additionally, the subcutaneous injection group exhibited greater body weight loss compared to the oral infection group, though this difference was not statistically significant. Although the overall mortality rates were identical between the two infection routes, this indicates the route of infection had minimal impact on mortality. However, the delayed time-to-death in the oral infection group suggests that different infection routes can influence the onset of disease in goslings [12]. Oral administration may prolong the disease course due to initial viral effects on the gastrointestinal tract. Nevertheless, following replication within the host, the virus still caused a highly mortality rate. Therefore, the JA2485 strain not only impairs the growth and development of goslings but also results in significant mortality, posing a notable threat to the poultry industry.

### 3.5. Necropsy Symptoms and Pathological Sections

**Figure 4:**
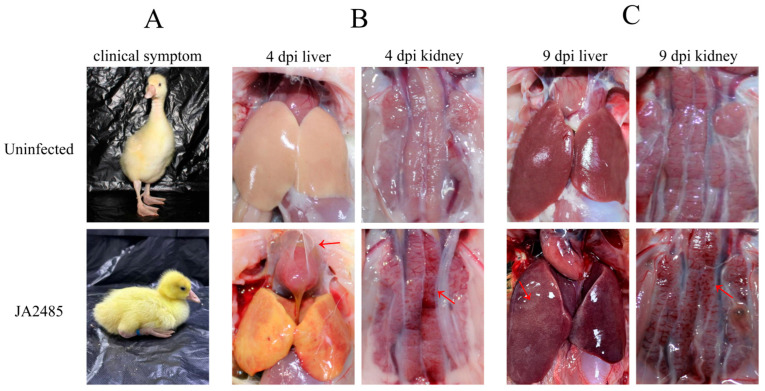
Clinical signs and post mortem lesions of 1-day-old Sanhua goslings infected with GoAdV-4 strain JA2485. Clinical signs of goslings in the subcutaneous injection group and oral infection group were observed and recorded after infection (**A**). Moribund goslings in the subcutaneous injection group at 4 dpi and the oral infection group at 9 dpi were selected and euthanized, respectively, for post mortem examination. Lesions in organs such as the liver (**B**) and kidney (**C**) were observed, with healthy goslings used as controls.

Following infection with the JA2485 strain, goslings in both challenge groups initially exhibited signs of weight loss. As body weight declined, these clinical manifestations were accompanied by behavioral changes such as increased recumbency and reduced appetite. Two to three days after the onset of weight loss, goslings began to display additional symptoms including depression, paralysis, and an inability to stand (Figure 4A). Most affected individuals lost their appetite entirely and succumbed to the disease at 2~3 dpi. Notably, goslings that experienced weight loss for one to two days followed by a gradual return to normal weight gain typically did not develop further illness or mortality.

The severity of clinical symptoms in the subcutaneous group peaked at 4 dpi, whereas those in the oral infection reached maximum severity at 9 dpi. Based on this observation, we selected moribund goslings from each group at the corresponding time points for necropsy. At 4 dpi, the livers of healthy goslings appeared pale yellow. In contrast, livers from the subcutaneous injection group exhibited classic pathological features of adenovirus infection compared to control group, including rounded margins, hepatomegaly, icterus, and focal hemorrhages. Pale yellow pericardial effusion was also observed within the pericardial sac (as shown by the red arrow). In the oral infection group, clinical signs were most pronounced at 9 dpi. Post mortem examination of affected goslings at the time point revealed hepatic rounding, swelling, and the presence of white necrotic foci (Figure 4B). Additionally, kidneys from gosling in both challenge groups displayed varying degrees of hemorrhagic lesions (Figure 4C).

### 3.6. Viral Load

**Figure 5:**
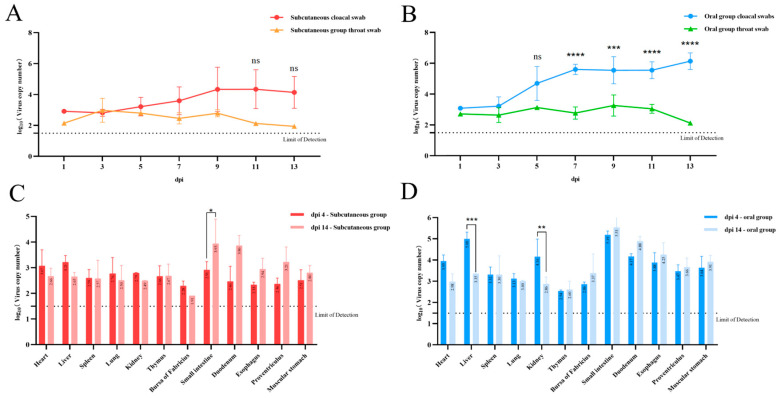
Viral load detection in 1-day-old Sanhua goslings infected with GoAdV-4 strain JA2485. Specific primers were designed based on the conserved 52K gene of adenoviruses to establish a real-time fluorescence quantitative PCR (qPCR) detection method, with a Limit of Detection (LOD) of 1 × 10^1.5^ copies/mL, and a plasmid DNA standard curve was constructed. Oropharyngeal and cloacal swabs were collected from goslings in the two infected groups at 1, 3, 5, 7, 9, 11, and 13 dpi, respectively (**A**,**B**). At 4 and 14 dpi, 3 goslings in each group were euthanized, and tissue samples such as the heart and liver were collected (**C**,**D**). DNA was extracted from swab and tissue samples after treatment, and viral load was quantified by qPCR. Data were presented as the mean ± standard deviation of viral copy numbers. * < 0.05, ** < 0.01, *** < 0.001, **** < 0.0001, indicating statistically significant differences between the groups.

Viral loads in throat and cloacal swabs from both challenge groups were quantified using qPCR (Figure 5A,B). The results showed that, following viral infection, cloacal swabs generally exhibited higher viral loads compared to throat swabs. In particular, although the difference was not statistically significant in the subcutaneous injection group, the oral infection group showed significantly higher viral loads in cloacal swabs than in throat swabs after 7 dpi. High viral loads were consistently detected in the cloacal swabs from both experimental groups. This may be attributed to the fact that during the later stages of infection, goslings primarily excreted the virus through the cloaca, resulting in greater viral accumulation in the anus. These findings suggest that the virus is more likely to spread with goose flocks via fecal excretion [13].

At 4 dpi, the subcutaneous injection group exhibited relatively uniform viral distribution across tissues, with slightly elevated levels observed in the liver and duodenum compared to other organs (Figure 5C). By 14 dpi, there was a general increase in viral load within the digestive tract organs, particularly in the duodenum, where the load was significantly higher than at 4 dpi. In the oral infection group, viral loads in all organs were generally higher than those in the subcutaneous group at 4 dpi and 14 dpi (Figure 5D). At 4 dpi, the highest viral loads in the duodenum and liver, followed by kidneys. However, by 14 dpi, viral loads in the liver and kidneys had significantly decreased compared to 4 dpi, while changes in other organs were not statistically significant.

The organ-specific viral load patterns observed in both experimental groups indicate that the liver and duodenum may be primary target organs for adenovirus infection in goslings. Furthermore, at 14 dpi, an increased level of viral shedding was observed in tissues associated with the digestive tract in both groups, suggesting that the virus disseminate within the flock through the gastrointestinal system following infection.

### 3.7. Histopathology

**Figure 6:**
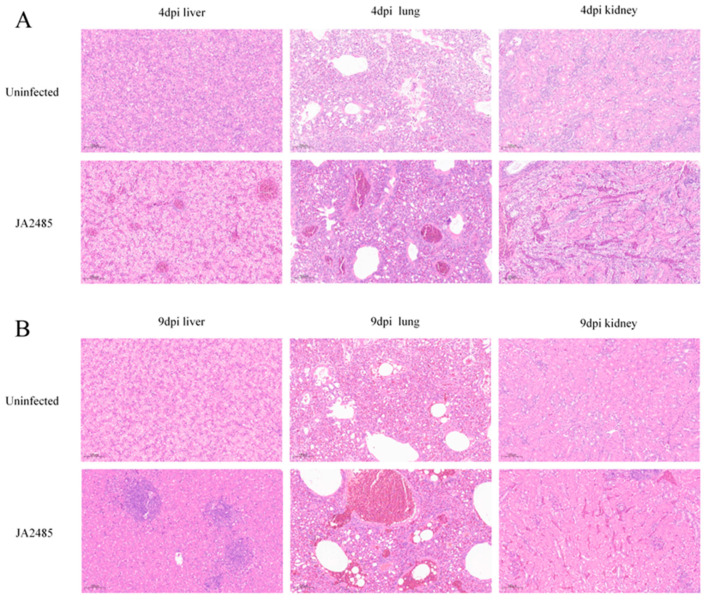
Representative histopathological lesions of 1-day-old Sanhua goslings infected with GoAdV-4 strain JA2485. At 4 dpi (subcutaneous injection group) (**A**) and 9 dpi (oral infection group) (**B**), goslings were euthanized, respectively, and tissues such as the liver, lung, and kidney were collected, with tissues from healthy goslings used as controls. Tissue samples were fixed in 4% formalin, embedded in paraffin, sectioned at 4–6 μm, and stained with hematoxylin and eosin (H&E). The histopathological characteristics were observed and photographed using a Nikon Eclipse E100 optical microscope (10× magnification).

Histopathological examination demonstrated that the livers of non-infected (control) goslings at 4 and 9 days post-infection (dpi) maintained a well-defined hepatic lobule structure with orderly arranged hepatocytes (Figure 6). In contrast, following infection with the JA2485 strain, liver tissues exhibited localized structural disorganization accompanied by focal inflammatory cell aggregation, which suggests early viral interference with the metabolic function of hepatocytes. Notably, the oral infection group at 9 dpi displayed extensive hepatic necrotic foci along with interstitial hyperplasia. The pulmonary architecture of uninfected goslings remained intact, with clearly demarcated alveolar structures. The subcutaneous injection group showed inflammatory cell infiltration and focal aggregation in lung tissue, while the oral infection group presented more severe histopathological alterations, including large vacuole-like structures in the lung parenchyma and clustered inflammatory cells, indicating potential impairment of respiratory function. Both infected groups exhibited progressive renal inflammation, characterized by increased inflammatory cell infiltration in the renal tubular interstitium, whereas the control group showed normal renal tubule-interstitial morphology. No detectable lesions were observed in the heart, spleen, bursa of Fabricius, thymus, or esophagus in either experimental group, indicating that both infection routes induce multi-organ damage involving the liver, lungs, and kidneys of goslings.

The severity of histopathological lesions was graded using a scoring system. The control group maintained a score of 0 throughout the experiment, with no histopathological damage, confirming the reliability of the experimental model. At 4 days post-infection (dpi), the subcutaneous injection group exhibited moderate damage in the liver, lung, and kidney (average score: 2.0), with lesions confined to local regions and no extensive structural disruption of the organs. At 9 dpi, the oral infection group showed significantly more severe multi-organ damage than the subcutaneous injection group (average score: 2.7): severe lesions (score: 3) were observed in the liver and lung, while moderate lesions (score: 2) were noted in the kidney—with a larger range of renal tubular necrosis compared to the subcutaneous injection group. Collectively, these results indicate that infection with GoAdV-4 strain JA2485 induces significant multi-organ pathological damage in goslings, with the liver and lungs serving as the primary target organs. The lesion score and severity in the 9 dpi oral infection group were higher than those in the 4 dpi subcutaneous injection group, suggesting that both the infection route and duration collectively influence the severity of pathological damage.

## 4. Discussion

In recent years, avian adenovirus outbreaks have become increasingly prevalent globally, leading to considerable economic losses in the poultry industry [14,15,16]. A variety of novel serotypes, such as FAdV-8a [17], FAdV-8b [18], DAdV-2 [19], DAdV-3 [20], DAdV-4 [21], PiAdV-1 [22], PsAdV-2 [23], have been identified across multiple avian species. These viruses continue to undergo genetic recombination and evolution, presenting significant challenges for disease prevention and control [24,25,26]. Given the aquatic behavior, domestic waterfowl are particularly effective vectors for adenoviruses transmission through contaminated water sources, thereby accelerating viral spread [27]. In this study, a GoAdV-4 strain was isolated from a three-week-old Sanhua goose and designated as JA2485 strain. Comprehensive investigations were carried out regarding its genetic evolution, in vitro propagation, and pathogenic characteristics.

Compared with the only 2 GoAdV-4 strains deposited in the NCBI database, the G + C content of the JA2485 strain determined in this study was 44.4%. For comparison, the G + C content of strain P29 was 44.7%, and that of strain CH-FJZZ-202201 was 44.5% [6,8]. Strain JA2485 showed the highest genomic similarity (98.6%) with strain CH-FJZZ-202201, both originating from China and isolated within a similar timeframe. Although the P29 strain was isolated from Hungary during the 1970s, it still exhibited a high similarity of 98.7% to JA2485. Phylogenetic analysis demonstrated that JA2485, together with P29 and CH-FJZZ-202201, formed distince evolutionary branches with comparable genetic distances across the whole genome and major structural proteins. This indicates that GoAdV-4 maintains intraspecific conservation. Despite its phylogenetic proximity to DAdV, JA2485 exhibits low sequence identity with DAdV and other avian adenovirus subtypes, further supporting the notion that GoAdV-4 follows a unique evolutionary pathway, potentially driven by host specific adaptation. These findings clarify the taxonomic status and evolutionary lineage of GoAdV-4, offering valuable insights into its origin.

Goose-derived viruses were typically propagated using duck or goose embryos at specific development stages [28]. Following this methodology, JA2485 strain was successfully cultured in 11- to 13-day-old goose embryos, yielding an EID_50_ titer of 10^2.2^/0.1 mL. Subsequently, animal challenge experiments were conducted by inoculating 1-day-old goslings via subcutaneous injection and oral administration. Both groups exhibited a mortality rate of 50%. Notably, disease onset occurred earlier following subcutaneous injection, likely due to direct viral entry into the circulatory system, which accelerates pathogenesis. Additionally, according to information from the goose farm where the virus was isolated, the JA2485 strain primarily infects 2- to 3-week-old goslings in practical aquaculture, causing a mortality rate of approximately 6% to 8%. In contrast, the challenged goslings in this experiment had a relatively higher mortality rate, which may be attributed to multiple variables differing between practical aquaculture settings and experimental challenge conditions, such as environmental factors, feeding management, and viral exposure dose.

The mortality profile of GoAdV-4 differs distinctly from that of other FAdV and DAdV. Specifically For young hosts, FAdV-1 demonstrates significantly reduced pathogenicity in chicks, eliciting only mild respiratory signs and a mortality rate typically below 10% [29]. In contrast, FAdV-4 induces a relatively high mortality rate ranging from 40% to as much as 100% in chicks [30,31], although a marked decline in mortality is observed in adult chicken post-infection. Among DAdV, DAdV-1 causes a mortality rate of merely 2–7% in ducklings and goslings [32], with the majority of infections remaining asymptomatic-this presents a stark contrast to the high pathogenicity of GoAdV-4 in goslings. Notably, certain specific strains of DAdV-3 (e.g., strain TZ193) are exceptions; these strains can induce a mortality rate as high as 100% in 5-day-old domestic ducklings [33], exhibiting strong pathogenicity analogous to GoAdV-4 but with distinct host specificity.

Post mortem examination revealed typical adenoviral lesions in the subcutaneous infected group, including pericardial effusion and hepatic changes characterized by yellowish discoloration and rounded liver edges [34]. In contrast, oral inoculation resulted in white punctate necrotic foci in the liver, consistent with previously reported GoAdV-induced hepatic pathology [35]. GoAdV-4 exhibits a consistent trait of inducing hepatic injury across different infection routes, a feature that aligns with avian adenoviruses, which prioritize the liver as their core target organ. Additionally, elevated viral concentrations were detected in the small intestine and duodenum, suggesting a predilection for viral replication within the gastrointestinal tract and potential cloacal excretion. Its abilities of colonization, replication, and subsequent excretion in the gastrointestinal tract are consistent with the common trait of avian adenoviruses and duck adenoviruses: these viruses are isolable from intestinal tissues or samples and primarily excrete via the intestinal tract [36,37]. This observation reflects the biological commonality of avian adenoviruses relying on the digestive tract for horizontal transmission. These findings enhance our understanding of GoAdV-4’s pathogenesis and transmission dynamics.

## 5. Conclusions

This study established a reliable method for cultivating the GoAdV-4 JA2485 strain in goose embryos, contributing to existing isolation and propagation protocols. Genetic analyses confirmed significant divergence between GoAdV-4 and other avian adenoviruses at both the whole-genome and structural protein levels. Current GoAdV-4 strains exhibit relative genetic stability without notable evolutionary shifts. Furthermore, animal experiments demonstrated that GoAdV-4 impairs gosling growth and development, induces high mortality, and poses a considerable risk of fecal–oral transmission. These results provide critical data for the formulation of targeted prevention and control strategies against GoAdV-4.

## Figures and Tables

**Table 1:** Primers used in this study for detection of the viruses.

Annealing Temperature	Upstream Primer (5′−3′)	Downstream Primer (5′−3′)	Product Size (bp)
FAdV-507	AATTTCGACCCCATGACGCGCCAGG	TGGCGAAAGGCGTACGGAAGTAAGC	507
GAst	AGTGGGACTCTGAAGAGG	GCTATAAGTTGCTCTGCAC	661
GPV	AGACTTATCAACAACCAYTG	TCACTTATTCCTGCTGTAG	780
GRV	TGAGACGCCTGACTACGATT	ATGCTTGGAGTGAGACGACT	380
MDPV	AAGAGAAAGAAAACCCGT	GTTGCCTCCAAAGGAGGTA	624
AIV-H5	GCCATTCCACAACATACACCC	CTCCCCTGCTCATTGCTATG	219
AIV-H7	CCCAATGTGAYCAATTCCT	GCTCCATTRGTTCTTATTCC	184
AIV-H9	GAATCCAGATCTTTCCAGAC	CCATACCATGGGGCAATTAG	383
NDV	ATGGGCYCCAGAYCTTCTAC	CTGCCACTGCTAGTTGTGATAATCC	536
IBDV	TGCAACAGCCAACATCAACG	GATCGTCACTGCTAGGCTCC	574

**Table 2:** Primers used to amplify the complete genomic sequence of strain JA2485.

Primer	Amplified Fragment Location (bp)	Upstream Primer (5′−3′)	Downstream Primer (5′−3′)
F1	1–1681	CATCATCATATATAAAATAACCACA	CATCGGTCAAATAGTCACAT
F2	1441–3361	TATTTCAAGTGCTTCGTGACGCTTT	CTGTTCAAATCCGCTATCGCTTATG
F3	3181–5101	TCTGGTTCGTTTCTTTCGGCTTCTG	ACGGACCTATAGCGATTGTCATGGA
F4	4741–6781	TTGTTGTAGGAAAGCAAAATAGGAT	GGAAGACGAGGAAGAAGAGGATACC
F5	6421–8521	AGTGCCTCAGATGGTGCTTCTAATA	CTGAAAAACCCCCGAGATTTGCCCT
F6	8221–10,321	TATGTTGTGTCCGATGACGATGATG	GTAGACTTAGAACCAGGAGACGCAG
F7	10,021–12,061	CGACTTCCATTTCTTCCTCTTCTTC	ATGTAAGCCATCTCTATCAATTCCC
F8	11,881–14,101	AAGACCGAAACGCTTGGTTGAGGGA	CTAAGTTCAGAAAATCCCGTACCCG
F9	16,781–17,341	TGCGTCGTTCAAGGTCCAGATCTAC	CTGCCTGTCATACTATCCAGTCGGG
F10	17,041–18,841	CTTGGAGGACCCGACTGGAT	ATCCACTATATTGGTCAATGGCACA
F11	18,421–20,341	AGACACAGTAAGTGCGTATGCGTTT	GCGGAAGAGGCTAATCTAGGGTTGA
F12	20,101–21,961	CCGAGAGAAATGTGCTTGCTATGGC	TTGAATGCCGTATGGGCTAAGATGG
F13	21,781–23,701	GGAAAAGGTGCATCTAAATTCGGGA	GTCCTCAACGACCACTCCGACTCTC
F14	23,461–25,321	CCTGTTCGTATTCCTCCTCGCTGTC	TCTATCCTCCTTTCGTAACGCCTAA
F15	25,021–27,001	GTTTATTCGGACCAAAGGCAAGGGA	GGGAGCCTTTTAGCGAGACGGAACT
F16	26,821–28,501	TTACCACCCTCTGTCTATATTCCAA	TTTAGAAGATTTTTTGGATGGACCG
F17	28,081–30,061	CATCTTTCGCTGGAACTAACGCTAA	GACTATCGGATCGTCTAACGGGGTG
F18	29,821–31,741	GACATGGACACCCTCCTCTCCCCTA	AGTTTTCGCTTTTTCACCCACGGAT
F19	31,501–33,481	GATACTCGACCTTTCGGCTGCCTTC	TTTCATAAAGTTTGCCACACTGCCA
F20	33,181–35,161	ATACAAAAGCAAAAATAGAAGCCCC	TCTTGCTTGTTCTTACTTGCTGCTT
F21	34,921–36,841	ATGAGAGATACGTTTTGTAACTTGG	ATGTTACTTCAATGGACAAGAGATA
F22	36,601–38,581	TGAGATTGACGCACTCTATCACCCA	ATGGTTAGTGGAGCATTGCTGGTAG
F23	38,341–40,201	GAAACTGTCCTCAAATGAAACCACT	AACCCTTCTTGTCTCCGCCCTCTCT
F24	39,961–41,941	TAGAGAGGGCGGAGACAAGAAGGGT	CAAATGGTTTACTTATGACCGATGT
F25	41,281–43,030	ATTCTCCTCCTTCCAGTTACCTATT	CATCATCATATATAAAATAACCACA

## Data Availability

The complete genome sequence of Goose Adenovirus Type 4 (GoAdV-4) strain JA2485 has been deposited in GenBank under accession number PQ152938 (publicly accessible at https://www.ncbi.nlm.nih.gov/genbank/ (accessed on 4 August 2024)).

## Abbreviations

The following abbreviations are used in this manuscript:

GoAdV-4	Goose Adenovirus Type 4
EID_50_	50% Embryo Infectious Dose
PCR	Polymerase Chain Reaction
FAdV	Fowl avianadenoviruses
GAst	Goose astrovirus
GPV	Goose parvovirus
GRV	Goose reovirus
MDPV	Duck parvovirus
AIV	Avian Influenza Viruses
NDV	Newcastle Disease Virus
IBDV	Infectious Bursal Fabricius Virus
GEF	Goose Embryo Fibroblasts
ITR	Inverted Terminal Repeat
qPCR	Quantitative PCR
dpi	Days Post Infection
H&E	Hematoxylin and Eosin
ORF	Open Reading Frame
LMH	Leghorn Male Hepatocellular (Cells)
